# Dietary Supplementation with Chromium DL-Methionine Enhances Growth Performance of African Catfish (*Clarias gariepinus*)

**DOI:** 10.1155/2023/7092657

**Published:** 2023-01-24

**Authors:** Frederik Kaiser, Michael Schlachter, Carsten Schulz, Claudia Figueiredo-Silva

**Affiliations:** ^1^Institute of Animal Breeding and Husbandry, Department of Marine Aquaculture, Christian-Albrechts-University Kiel, Olshausenstraße 40, 24098 Kiel, Germany; ^2^Fraunhofer Research Institution for Individualized and Cell-Based Medical Engineering, Aquaculture und Aquatic Resources, Hafentörn 3, 25761 Büsum, Germany; ^3^Zinpro Corporation, 10400 Viking Drive, Eden Prairie, MN 55344, USA

## Abstract

Sustainable aqua feeds have become an urgent necessity for future-oriented aquaculture sector development, and especially mineral supply could be limited when diets are being prepared with low amounts of animal-based sources. Since knowledge about the efficiency of organic trace mineral supplementation in different species of fish is limited, the effects of chromium DL-methionine in African catfish nutrition were evaluated. Four commercially based diets with increasing chromium DL-methionine supplementation (0, 0.2, 0.4, and 0.6 mg Cr kg^−1^) in the form of Availa-Cr 1000 were fed to African catfish (*Clarias gariepinus* B., 1822) in quadruplicate groups for 84 days. Growth performance parameters (final body weight, feed conversion ratio, specific growth rate, daily feed intake, protein efficiency ratio, and protein retention efficiency), biometric indices (mortality, hepatosomatic index, spleen somatic index, and hematocrit), and mineral retention efficiency were assessed at the end of the feeding trial. The specific growth rate was significantly increased in fish-fed diets with 0.2 mg Cr kg^−1^ and 0.4 mg Cr kg^−1^ supplementation in comparison with control and based on the second-degree polynomial regression analysis; supplementation with 0.33 mg Cr kg^−1^ was optimal in commercially based diets for African catfish. Chromium retention efficiency was reduced with increasing supplementation levels; however, the chromium content of the whole body was comparable to literature. The results suggest that organic chromium supplementation is a viable and safe supplement for diets to increase the growth performance of African catfish.

## 1. Introduction

Feed is one of the highest cost factors in fish farming, and it has become evident that the utilization of diets for aquatic species has to be as efficient as possible to reduce pollution of the environment [1]. Decade-long research has demonstrated that supplementation of aqua feeds with different nutrients, vitamins, or minerals can be beneficial for fish health, growth, and overall feed efficiency, especially for diets low in fish meal [2].

Chromium is an essential mineral for humans and certain animals [3], although an essentiality could not be demonstrated in fish based on the definition of an essential trace element [4]. However, dietary supplementation with Cr in fish diets resulted in enhanced growth performance as well as improved immune response and stress sensitivity in numerous species of fish [5–15]. Especially trivalent chromium (Cr^+3^) can support the metabolism of carbohydrates, lipids, and proteins by elevating the activity of digestive enzymes [16] and potentiating the action of insulin [3]. These mechanisms can lead to increased energy and protein utilization and subsequently improved growth performance of fish [8]. However, high amounts of Cr in diets can lead to toxic effects like interrupting cellular integrity and altering several hematological indices [6, 17], and the health status of fish should be monitored when including additional sources of Cr in fish diets. Generally, organic chromium sources are more bioavailable in comparison with inorganic chromium [3] and were therefore preferentially included in aqua feeds. Among different organic sources of chromium, dietary chromium methionine (CrMet) supplementation was recently demonstrated to be nontoxic for fish even at high doses (2 mg kg^−1^) and to result in improved growth rate and feed efficiency in comparison with chromium oxide and chromium picolinate [18].

Effects on growth performance and health status of fish varied depending on the form and dose of Cr, as well as on experimental duration and fish species [6]. The aim of this study was therefore to investigate the impact of CrMet supplementation in the form of Availa-Cr 1000, a commercially available product containing a chromium DL-methionine complex, on growth performance and health status of African catfish after 84 days of feeding. To enable the highest level of relevance towards a practical application, a commercially based diet with low fish meal content was supplemented with gradually increasing levels of the product under review. African catfish were selected for this study as they are of great importance to the global aquaculture sector [19].

## 2. Materials and Methods

### 2.1. Ethical Considerations

The research site adhered to the guidelines set out in the German Animal Welfare Act and was supervised by an animal welfare officer. The registration number of the trial at the ministry was V242 – 32390/2021.

### 2.2. Experimental Setup

800 mixed-sex African catfish (54.4 ± 1.2 g, Aquaculture ID, Nederweert, Netherlands) were adapted to the recirculating system of the Fraunhofer Research Institution for Individualized and Cell-Based Medical Engineering, Büsum, Germany, for 7 days. During adaptation, fish were fed a commercial catfish diet daily at 2% of their body weight (Aller CLARIA FLOAT 4.5 mm; 42 g 100 g^−1^ crude protein, 12 g 100 g^−1^ crude fat, 29.5 100 g^−1^ NfE, 5.6 100 g^−1^ ash, 2.9 100 g^−1^ fiber, and 20.2 MJ kg^−1^ gross energy; Aller Aqua A/S, Christiansfeld, Denmark) before being starved for two days before the experimental start. Subsequently, quadruplicate groups of catfish consisting of 50 individuals were assigned to 16 aquaria (60 l) in a semirandomized manner to avoid potential tank effects. The initial stocking density was set to 50 kg m^−3^. After 6 weeks, the tank volume was doubled by pulling a bulkhead.

The aquaria were embedded in a recirculating system (15 m^3^, water turnover rate 4 h^−1^) including a drum filter (mesh size 20 *μ*m), moving bed biofilter (6 m^3^), and UV treatment (3 × 100 W). Water parameters were measured daily to ensure optimal rearing conditions during the growth trial. Oxygen saturation was at 103.9 ± 6.1% (Handy Polaris; Oxy-Guard International A/S, Birkerod, Denmark), the temperature at 27.4 ± 0.9°C, and pH at 7.4 ± 0.3 (GMH 5550, Digital pH-/mV-/Thermometer, Greisinger Electronic, D). Total ammonia nitrogen remained below 0.4 mg l^−1^ and total nitrate nitrogen below 1 mg l^−1^ (Microquant test kit for NH_4_ and NO_2_; Merck KgaA, Darmstadt, Germany). The photoperiod was set to 24 h of darkness. Red light headlamps were used to check the mortalities and health status of fish during the experiment.

African catfish were fed twice daily (9:00 am and 3:00 pm) to apparent satiation for 84 days. Additionally, a fixed rate of 0.5% of body weight was fed during night hours by automated belt feeders to reduce aggression among fish. To calculate the exact feed intake, an average pellet weight was measured, and excess pellets were collected after the feeding events.

### 2.3. Experimental Diets

Four diets were prepared for the feeding trial (CRTL = control, CR 1 = chromium diet 1, CR 2 = chromium diet 2, and CR 3 = chromium diet 3). A diet reflecting typical commercial catfish feeds in ingredient and nutrient composition served as control (Tables 1 and 2), while the remaining diets were copies of the control, supplemented with increasing levels of Availa-Cr 1000 (200, 400, and 600 mg kg^−1^, Table 3). Extruded floating pellets were produced by SPAROS, Olhão, Portugal. The analyzed chromium content of the control diet was 2.37 mg kg^−1^, and the content of supplemented diets exceeded expectations (Tables 3 and 4). However, the analyzed chromium content of diets was still within relevant doses, and a continuous increase in supplementation level persisted among experimental diets. The evaluated product was chromium DL-methionine (Availa-Cr 1000) with batch number HPA19122, provided by Zinpro Corporation in a ready-to-use powder form. The content of chromium methionine in the product was 2 g 100 g^−1^. The analyzed chromium content of the product was 1180 mg Cr kg^−1^.

### 2.4. Sampling

The experiment and fish sampling were conducted according to the German Animal Welfare Act as amended 2016 and according to the German Animal Welfare Laboratory Animal Regulation. The trial was registered as number V242 – 32390/2021 at “Ministerium für Energiewende, Landwirtschaft, Umwelt, Natur und Digitalisierung”.

All fish were starved for 48 hours before sampling. After the initial adaptation phase as well as after 84 days of feeding, three fish per tank were sampled for determination of whole body composition, trace mineral content, hepatosomatic index (HSI), spleen somatic index (SSI), and hematocrit. Fish were anesthetized with a blunt hit on the central nervous system and subsequently euthanized by piercing the heart. Whole body samples were stored at -20°C until being homogenized and freeze-dried (Alpha 1-2 LD plus, Christ, Osterode, Germany) before analysis. For the calculation of hematocrit, blood was sampled with syringes at the caudal vein and subsequently centrifuged (Haematokrit 210, Andreas Hettich GmbH & Co. KG, Tuttlingen, Germany). Values for hematocrit were determined optically with the scale attached to the centrifuge.

Growth performance parameters were calculated including feed conversion ratio (FCR = g feed intake/g weight gain), specific growth rate (SGR = (ln (FBW)–ln (IBW))/feeding days^∗^ 100), daily feed intake (DFI = FCR^∗^SGR), protein efficiency ratio (PER = g body weight gain/g crude protein intake), and protein retention efficiency (PRE = g crude protein gained/g crude protein intake^∗^100).

### 2.5. Analysis of Nutrients and Trace Minerals

Analysis of macronutrients in diets and the whole body was carried out according to the European Commission Regulation (EC) No. 152/2009 [20]. Analysis of diets and homogenized whole bodies was carried out in duplicates. For the determination of crude lipid content in the whole body, methods according to the Soxhlet protocol (Soxtherm, C. Gerhardt GmbH & Co. KG, Königswinter, Germany) were applied. Crude protein content was determined using standard Kjeldahl methods. Before the determination of ash content via a combustion oven (P300, Nabertherm, Lilienthal, Germany) at 550°C for 12 hours, the dry matter content of samples was ascertained by drying (ED 53, Binder GmbH, Tuttlingen, Germany) for 4 hours at 103°C. Nitrogen-free extracts plus crude fiber content were defined as the remaining portion of macronutrients in diets and the whole body.

Trace minerals (Cr, Cu, Fe, Zn, Mn, and Se) in diets and whole body were analyzed by an external lab (Agrolab LUFA, Kiel, Germany) following the standards for trace mineral analysis [21, 22].

### 2.6. Statistical Analysis

The statistical analyses were performed using SPSS 21 for Windows (SPSS Inc., Chicago, U.S.). Data are presented as mean ± standard deviation (SD) for each treatment and comparisons between treatments. Before the application of one-way analysis of variances (ANOVA), Kolmogorov-Smirnov and Levene tests were applied to determine normal distribution and homogeneity of variances. Tukey's HSD test was used for multiple comparisons when differences among groups were identified. The aggregate type I error was defined at 5% (*P* < .05) for each set of comparisons to determine statistical significance.

Additionally, regression analysis between the calculated organic Cr supplementation level as the independent variable and SGR as the dependent variable was calculated by polynomial regressions. The optimum dosage was calculated for slope = 0.

## 3. Results

### 3.1. Growth Performance

After 84 days of feeding, growth performance was significantly affected by CrMet supplementation. Final body weight and SGR were significantly increased in dietary groups CR 1 (683.94 ± 44.91 and 3.03 ± 0.06) and CR 2 (670.52 ± 32.92 and 2.99 ± 0.06) compared to control (591.21 ± 17.15 and 2.83 ± 0.03; Table 5). The remaining growth performance parameters were at a similar level among dietary treatments.

**Figure 1:**
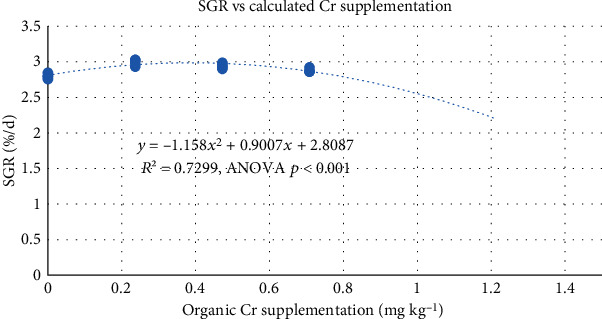
Regression analysis with specific growth rate as dependent variable and organic chromium supplementation as the independent variable.

The second-degree polynomial regression of dietary organic chromium supplementation and SGR was highly significant (*P* < 0.001, Figure 1), which allowed a calculation of the optimal chromium content by performing the first derivation of the equation and calculating *x* for *f* (*x*) = 0. The resulting optimal dietary organic chromium supplementation level was 0.39 mg kg^−1^ (0.33 mg Cr kg^−1^ from CrMet).

### 3.2. Body Composition

No significant differences between dietary groups were detected in proximate whole body composition (Table 6), trace mineral content of whole body (Table 7), or trace mineral retention (Table 8) after 84 days of feeding.

### 3.3. Biometric Indices

No significant differences were observed in any of the biometric indices investigated in this study (Table 9).

## 4. Discussion

In the present day, sustainable aqua feeds appear to be an inevitable necessity, and feed additives could contribute significantly by improving the effective utilization of feed ingredients. The present study demonstrated that the supplementation of diets with CrMet could improve the growth performance of African catfish. This effect could have multiple rationales. Firstly, organic minerals are more readily available for fish compared to their inorganic counterparts [3, 9]. Secondly, dietary chromium supplementation has been demonstrated to support the immune response [13] and reduce the stress level of fish [8, 12]. Lastly, diets with chromium addition can improve the energy metabolism of fish by activating various digestive enzymes and enhancing the activity of insulin [2, 3, 8, 10, 18].

According to the results of the current study, dietary organic chromium supplementation appears to have a minor positive effect on both feed conversion and feed intake, which in turn resulted in a significantly improved growth rate of catfish. Multiple earlier studies demonstrated a positive effect on growth rates of various species of fish including hybrid tilapia (*Oreochromis niloticus* L., 1758 *x Oreochromis aureus* S., 1864; [14]; form of chromium supplemented: CrCl_3_6H_2_O, Na_2_CrO_4_4H_2_O, and Cr_2_O_3_), Nile tilapia (*Oreochromis niloticus*; [11]; Cr picolinate, 0.6-1.8 mg kg^−1^), grass carp (*Ctenopharyngodon idella* V., 1844; [23]; Cr picolinate, 0.2-3.2 mg kg^−1^), common carp (*Cyprinus carpio* L., 1758; [8, 18]; Cr methionine, 0.31-3.64 mg kg^−1^; Cr oxide, Cr picolinate, and Cr methionine, 2 mg kg^−1^), mirror carp (*Cyprinus carpio*; *[*5*]*; Cr chloride, Cr picolinate, and Cr yeast, 0.5-2 mg kg^−1^), large yellow croaker (*Larimichthys crocea* R., 1846; [15]; Cr polynicotinate, 5-80 mg kg^−1^), blunt snout bream (*Megalobrama amblycephala* Y., 1955; [24]; Cr picolinate, 0.2-12 mg kg^−1^), snakehead (*Channa argus* C., 1842; [10]; Cr yeast, 200 mg kg^−1^), and striped catfish (*Pangasianodon hypophthalmus* S., 1878; [6]; Cr, 2-8 mg kg^−1^). However, other studies could not show any response from different species of fish to dietary Cr supplementation ([25]; Cr picolinate, 0.8-1.2 mg kg^−1^; [26]; Cr yeast, 0.8 mg kg^−1^; [27]; Cr picolinate, 2 mg kg^−1^; [28]; Cr picolinate, 1.6 mg kg^−1^). These contradictory results can most likely be explained by different factors influencing the effects of dietary Cr, including form and dose of Cr, duration of experiment, and behavior of concerned species [6].

The low bioavailability of inorganic chromium is caused by a multitude of factors including the formation of nonsoluble Cr oxides, binding to natural chelate-forming compounds in feeds, and interference with ion forms of other minerals [3]. Results suggest that supplementation with organic CrMet is beneficial for the nutritious value of commercially based diets for African catfish. This positive effect is in accordance with Cui et al. [18], who demonstrated that dietary CrMet supplementation is superior in comparison with inorganic Cr and Cr picolinate in common carp due to increased absorption efficiency. Dietary CrMet supplementation also improved the growth performance of common carp in earlier research [8] and of different crustacean species [29, 30]. Additionally, organic-chelated minerals could provide a complex that is more stable in the upper digestive tract in comparison with mineral salts, thereby increasing the bioavailability of the minerals [31]. According to Pechova and Pavlata [3], the absorption efficiency of inorganic Cr^+3^ is inversely proportional to the dietary level. A similar trend was also observed in the present study with organic Cr supplementation, somewhat contradicting observations from Cui et al. [18]. However, the analyzed content of Cr in diets differed from the expected values from organic supplementation (Tables 3 and 4), which was most likely caused by contamination with inorganic Cr during feed production. These elevated levels of inorganic Cr could explain the reduced absorption efficiency of Cr. Despite a trend toward reduced absorption of Cr at higher supplementation levels, the content of Cr in the whole body is still comparable to the results of other literature [5].

Due to handling, fish might have been exposed to short periods of stress during the experiment. Stress can increase the demand for Cr in humans and animals [3]. The stress-related secretion of cortisol, which acts as an antagonist for insulin, elevates the blood glucose concentration. Latter elevation results in the mobilization and subsequent excretion of Cr [32]. Multiple studies have shown a reduced sensitivity to different stressors due to dietary supplementation with Cr in various animals including fish [7, 12, 33–35]. This reduced sensitivity to stress can in turn elevate growth performance by enhancing energy utilization, absorption, and allocation [36]. Additionally, Risha et al. [13] demonstrated that Cr supports the nonspecific immunity in Nile tilapia, which was also demonstrated in other animals [37–39]. Furthermore, Cr supplementation also supported the antioxidative status of Nile tilapia [13]. The immune response and antioxidant status could therefore be enhanced even without stress [13]. Hence, supplementation with bioavailable Cr could result in increased growth performance by reducing stress sensitivity, as well as improving immune response and antioxidant status.

Chromium is also involved in the activation of digestive enzymes and protein stabilization, which are primary steps for the metabolism of carbohydrates, proteins, and lipids [40]. Supplementation with Cr has been shown to improve the activity of glycolytic and lipogenic enzymes in the liver of common carp [8, 16]. Additionally, Cr acts as a cofactor for insulin, enhancing its activity, which potentiates the regulation of glycemia and muscle protein deposition [3]. Increased serum insulin concentrations have been observed previously in fish at a Cr supplementation level of 0.8 mg kg^−1^ [23], and the anabolic role of this hormone resulted in improved growth performance. Additionally, the enhanced glucose clearance from blood due to higher insulin activity [2] can improve feed intake, since reduced glycemia has been demonstrated to increase feed consumption [41, 42]. Since no analysis of digestive enzymes was conducted, a significant effect was not demonstrated during this study; however, improved activation of digestive enzymes could have contributed to the overall significant effect on growth performance. The overall improvement in feed conversion and feed intake was of a minor extent, indicating an effect of a lesser degree from Cr supplementation on each growth factor. This small effect could partly be explained by diet formulation since the objective of this study was to show the effects of a diet with high relevancy to the industry. The commercially based diet used in this study has a comparatively high crude protein content, which could reduce the effectiveness of a protein-sparing effect from improved carbohydrate and lipid metabolism. This would also be a tangible explanation for the similar protein retention between all dietary groups. The latter observation was contrary to earlier research results demonstrating improved protein retention due to dietary supplementation with Cr in different species of fish [6, 23]. Additionally, the effects of dietary Cr supplementation were more prominent when glucose was included in diets for fish in comparison to starch [14, 43, 44]. More complex carbohydrates tend to lead to a less intense blood glucose peak [2]. Therefore, effects on feed intake due to improved clearance of glucose from the bloodstream could be less prominent considering the current feed formulation in this trial. Despite diet formulation, CrMet was still able to improve the growth performance of African catfish significantly, and it can be expected that this effect would be greater in diets containing less complex carbohydrates or lower amounts of protein. These effects have already been demonstrated in literature for different species of fish [6, 14, 23, 43, 44].

High amounts of Cr supplementation can have toxic effects on fish [17]. Akter et al. [6] observed changes in hematological indices, indicating toxic effects for striped catfish at high dietary Cr content (8 mg kg^−1^). Based on the results of the current study, supplemented levels of CrMet were not toxic for African catfish up to a dietary supplementation level of 0.6 mg Cr kg^−1^. This is in line with the findings from Akter et al. [6] for striped catfish. Additionally, no significant differences were observed in other biometric indices (Table 9), indicating that no negative effects on the liver and spleen occurred due to the supplementation with CrMet. However, it should be noted that the analysis of health parameters was not the main focus of this study and additional conformation for the safe application of CrMet should be collected in future trials with a more comprehensive amount of analyzed health parameters.

Our results demonstrated that supplementing a commercially based diet containing 2.37 mg Cr kg^−1^ with 330 mg kg^−1^ CrMet (0.39 mg Cr kg^−1^) optimizes the growth performance of African catfish. A similar value has been determined for striped catfish (2.82 mg Cr kg^−1^ total dietary supply; [6]), Nile tilapia (3.49 mg Cr kg^−1^ total dietary supply; [45]), and grass carp (0.8 mg Cr picolinate kg^−1^ supplementation; [23]).

## 5. Conclusion

Supplementing commercially based diets of African catfish with CrMet significantly improved growth performance at dietary organic Cr supplementation levels of 0.2 and 0.4 mg kg^−1^. Based on regression analysis, 0.33 mg Cr kg^−1^ from CrMet supplementation in commercially based diets for African catfish was optimal.

## Figures and Tables

**Table 1:** Feed ingredients of control diet.

Ingredient (g 100 g^−1^)	
Fish meal 60	7.50
Poultry meal	7.50
Soy protein concentrate	10.00
Wheat gluten	4.00
Corn gluten meal	15.00
Soybean meal	22.50
Sunflower meal 40	5.00
Wheat meal	10.45
Wheat bran	5.00
Vitamin premix 1%^1^	1.00
Mineral premix ZINPRO 2%^2^	2.00
Vitamin E50	0.05
Antioxidant	0.20
Monoammonium phosphate	0.70
L-lysine HCL 99%	0.60
Fish oil	2.00
Rapeseed oil	6.50

^1^Vitamin premix (IU/mg kg^−1^ diet, PREMIX Lda., Neiva, Portugal): DL-alpha tocopherol acetate, 100 mg; sodium menadione bisulfate, 25 mg; retinyl acetate, 20,000 IU; DL-cholecalciferol, 2,000 IU; thiamin, 30 mg; riboflavin, 30 mg; pyridoxine, 20 mg; cyanocobalamin, 0.1 mg; nicotinic acid, 200 mg; folic acid, 15 mg; ascorbic acid, 500 mg; inositol, 500 mg; biotin, 3 mg; calcium pantothenate, 100 mg; choline chloride, 1,000 mg, betaine, 500 mg; excipient wheat middlings. ^2^Mineral premix (mg kg^−1^ diet, PREMIX Lda., Neiva, Portugal): zinc from Availa®Zn 100, 80; selenium from Availa®Se 2000, 0.2; manganese from Availa®Mn 80, 30; iron from Availa®Fe 90, 100; copper from Availa®Cu 100, 10; excipient wheat.

**Table 2:** Crude nutrient and gross energy content of diets (mean ± SD).

Diet	Dry matter (%)	Ash (% DM)	Crude protein (% DM)	Crude fat (% DM)	NfE^1^ (% DM)	Energy (MJ/kg DM)
CTRL	92.43 ± 0.07	6.35 ± 0.05	48.02 ± 0.03	13.59 ± 0.05	32.05	22.12 ± 0.04
CR 1	92.90 ± 0.03	6.31 ± 0.02	47.77 ± 0.02	13.31 ± 0.01	32.61	22.11 ± 0.01
CR 2	92.46 ± 0.04	6.33 ± 0.03	47.81 ± 0.01	13.39 ± 0.07	32.47	22.17 ± 0.03
CR 3	92.72 ± 0.00	6.35 ± 0.01	47.94 ± 0.13	13.66 ± 0.04	32.05	22.14 ± 0.02

^1^NfE (nitrogen − free extract) + crude fiber = 100–(crude protein + crude lipid + crude ash). CR 1 = chromium diet 1; CR 2= chromium diet 2; CR 3 = chromium diet 3.

**Table 3:** Supplementation of diets with Availa-Cr 1000: supplemented and calculated levels of inclusion.

Diet	Availa-Cr 1000 inclusion in feed (mg kg^−1^)	Supplementation levels (mg Cr kg^−1^)	Calculated organic Cr levels in diet (mg kg^−1^)^1^
CTRL	0	0	0
CR 1	200	0.2	0.24
CR 2	400	0.4	0.47
CR 3	600	0.6	0.71

^1^Based on the analyzed amount of Cr in the product. CR 1 = chromium diet 1; CR 2 = chromium diet 2; CR 3 = chromium diet 3.

**Table 4:** Trace mineral content of the diets.

Diet	Chromium (mg kg^−1^)	Selenium (mg kg^−1^)	Copper (mg kg^−1^)	Iron (mg kg^−1^)	Zinc (mg kg^−1^)	Manganese (mg kg^−1^)
CTRL	2.37	0.86	27.8	197	104	57.4
CR 1	2.88	0.92	29.5	211	115	59.2
CR 2	3.33	0.96	29.5	222	119	60.1
CR 3	3.47	0.93	30.1	220	119	60.2

CR 1 = chromium diet 1; CR 2 = chromium diet 2; CR 3 = chromium diet 3.

**Table 5:** Growth performance parameter of African catfish after 84 days of feeding (mean ± SD).

Diet	IBW	FBW	FCR	SGR	DFI	PRE	PER
CTRL	54.98 ± 1.22	591.21 ± 17.15^a^	0.86 ± 0.01	2.83 ± 0.03^a^	2.43 ± 0.03	49.83 ± 1.01	2.65 ± 0.02
CR 1	53.67 ± 1.27	683.94 ± 44.91^b^	0.83 ± 0.02	3.03 ± 0.06^c^	2.51 ± 0.09	46.68 ± 2.60	2.68 ± 0.08
CR 2	54.35 ± 1.38	670.52 ± 32.92^b^	0.84 ± 0.01	2.99 ± 0.06^bc^	2.50 ± 0.07	49.29 ± 1.46	2.64 ± 0.04
CR 3	54.55 ± 0.77	634.01 ± 4.25^ab^	0.83 ± 0.01	2.92 ± 0.01^ab^	2.42 ± 0.05	49.94 ± 0.89	2.67 ± 0.05

IBW = initial body weight; FBW = final body weight; FCR (feed conversion ratio) = g feed intake/g weight gain; SGR (specific growth rate) = (ln (FBW)–ln (IBW))/feeding days^∗^ 100; DFI (daily feed intake) = daily feed intake in%body weight; PER (protein efficiency ratio) = g body weight gain/g crude protein intake; PRE (protein retention efficiency) = g crude protein gained/g crude protein intake^∗^100. Values in the same row with different superscript letters are significantly different (*P* < .05). CR 1 = chromium diet 1; CR 2 = chromium diet 2; CR 3 = chromium diet 3.

**Table 6:** Proximate whole body composition of fish after 84 days of feeding (mean ± SD).

Diet	Moisture (% OS)	Crude ash (% OS)	Crude protein (% OS)	Crude fat (% OS)	Gross energy (MJ kg^−1^ OS)
CTRL	69.61 ± 0.97	2.77 ± 0.10	17.09 ± 0.33	10.51 ± 0.76	8.10 ± 0.32
CR 1	69.72 ± 0.78	2.67 ± 0.10	17.07 ± 0.20	10.36 ± 0.72	8.13 ± 0.30
CR 2	68.58 ± 0.86	2.73 ± 0.06	16.98 ± 0.30	11.59 ± 0.72	8.60 ± 0.30
CR 3	68.81 ± 0.50	2.83 ± 0.12	17.04 ± 0.20	11.20 ± 0.32	8.49 ± 0.17

Values in the same row with different superscript letters are significantly different (*P* < .05). CR 1 = chromium diet 1; CR 2 = chromium diet 2; CR 3 = chromium diet 3.

**Table 7:** Trace mineral content of the whole body of fish after 84 days of feeding (mean ± SD).

Diet	Chromium (mg kg^−1^)	Selenium (mg kg^−1^)	Copper (mg kg^−1^)	Iron (mg kg^−1^)	Zinc (mg kg^−1^)	Manganese (mg kg^−1^)
CTRL	0.56 ± 0.30	1.13 ± 0.03	7.72 ± 7.69	45.05 ± 2.93	40.55 ± 5.40	9.68 ± 0.73
CR 1	0.52 ± 0.45	1.08 ± 0.05	20.32 ± 25.65	43.25 ± 6.86	48.63 ± 13.17	10.75 ± 0.99
CR 2	0.44 ± 0.18	1.15 ± 0.04	4.18 ± 0.98	44.88 ± 2.63	40.20 ± 2.49	10.15 ± 0.52
CR 3	0.34 ± 0.07	1.12 ± 0.00	5.32 ± 3.13	47.23 ± 1.18	41.58 ± 3.99	10.18 ± 1.14

Values in the same row with different superscript letters are significantly different (*P* < .05). CR 1 = chromium diet 1; CR 2= chromium diet 2; CR 3 = chromium diet 3.

**Table 8:** Trace mineral retention of African catfish after 84 days of feeding (mean ± SD).

Diet	Chromium retention (%)	Selenium retention (%)	Copper retention (%)	Iron retention (%)	Zinc retention (%)	Manganese retention (%)
CTRL	8.63 ± 4.86	40.88 ± 2.38	8.68 ± 10.97	45.05 ± 2.93	8.78 ± 2.12	5.55 ± 0.43
CR 1	6.58 ± 6.25	38.00 ± 1.72	25.91 ± 34.66	43.25 ± 6.86	11.82 ± 4.53	6.36 ± 0.55
CR 2	4.77 ± 2.28	37.75 ± 3.57	3.60 ± 1.41	44.88 ± 2.63	7.89 ± 0.44	5.62 ± 0.62
CR 3	3.34 ± 0.83	37.40 ± 0.67	4.91 ± 4.12	45.97 ± 2.29	8.14 ± 1.40	5.61 ± 0.80

Trace mineral retention (%) = trace mineral content whole body/trace mineral content diet. Values in the same row with different superscript letters are significantly different (*P* < .05). CR 1 = chromium diet 1; CR 2 = chromium diet 2; CR 3 = chromium diet 3.

**Table 9:** Biometric indices of fish after 84 days of feeding.

Diet	Mortality (%)	HSI (%)	SSI (%)	Hematocrit (%)
CTRL	2.5 ± 1.7	1.49 ± 0.28	0.08 ± 0.02	38.8 ± 4.4
CR 1	3.0 ± 2.2	1.61 ± 0.32	0.09 ± 0.03	35.2 ± 3.2
CR 2	4.0 ± 3.2	1.68 ± 0.44	0.07 ± 0.02	38.0 ± 2.9
CR 3	3.5 ± 2.2	1.70 ± 0.28	0.07 ± 0.01	36.9 ± 2.7

HSI (hepatosomatic index) = g liver weight/g fish weight^∗^ 100; SSI (spleen somatic index) = g spleen weight/g fish weight^∗^ 100; Hematocrit = %of blood cells in the blood. Values in the same row with different superscript letters are significantly different (*P* < .05). CR 1 = chromium diet 1; CR 2 = chromium diet 2; CR 3 = chromium diet 3.

## Data Availability

Data will be made available upon reasonable request.

## References

[1] FAO (2022). *The State of World Fisheries and Aquaculture. Towards blue transformation*.

[2] National Research Council (2011). *Nutrient Requirements of Fish and Shrimp*.

[3] Pechova A., Pavlata L. (2007). Chromium as an essential nutrient: a review. *Veterinary Medicine*.

[4] Lall S. P., Kaushik S. J. (2021). Nutrition and metabolism of minerals in fish. *Animals*.

[5] Ahmed A. R., Jha A. N., Davies S. J. (2012). The efficacy of chromium as a growth enhancer for mirror carp (*Cyprinus carpio* L): an integrated study using biochemical, genetic, and histological responses. *Biological Trace Element Research*.

[6] Akter S., Jahan N., Rohani M. F., Akter Y., Shahjahan M. (2021). Chromium supplementation in diet enhances growth and feed utilization of striped catfish (*Pangasianodon hypophthalmus*). *Biological Trace Element Research*.

[7] Castro M. P., Claudiano G. S., Petrillo T. R. (2014). Acute aerocystitis in Nile tilapia bred in net cages and supplemented with chromium carbochelate and *Saccharomyces cerevisiae*. *Fish & Shellfish Immunology*.

[8] Cui P., Yin S., Cheng Z., Qiao X., Zhou Q. (2018). Effects of dietary chromium methionine on growth performance, hematological characteristics and carbohydrate metabolic enzyme activities of common carp (*Cyprinus carpio*). *Israeli Journal of Aquaculture - Bamidgeh*.

[9] Gatta P. P., Thompson K. D., Smullen R., Piva A., Testi S., Adams A. (2001). Dietary organic chromium supplementation and its effect on the immune response of rainbow trout (*Oncorhynchus mykiss*). *Fish & Shellfish Immunology*.

[10] Hou Y., Hou Y., Yao L., Chen S., Fan J., Qian L. (2019). Effects of chromium yeast, tributyrin and bile acid on growth performance, digestion and metabolism of *Channa argus*. *Aquaculture Research*.

[11] Li H., Meng X., Wan W. (2018). Effects of chromium picolinate supplementation on growth, body composition, and biochemical parameters in Nile tilapia *Oreochromis niloticus*. *Fish Physiology and Biochemistry*.

[12] Liang H., Ge X., Xia D., Ren M., Mi H., Pan L. (2022). The role of dietary chromium supplementation in relieving heat stress of juvenile blunt snout bream *Megalobrama amblycephala*. *Fish & Shellfish Immunology*.

[13] Risha E., Ahmed F., Khaled A. A., Hossain F. M. A., Akhtar N., Zahran E. (2022). Interactive effects of dietary betaine and chromium picolinate on the immunomodulation, antioxidative response and disease resistance of Nile tilapia (*Oreochromis niloticus*). *Aquaculture Research*.

[14] Shiau S. Y., Chen M. J. (1993). Carbohydrate utilization by tilapia (*Oreochromis niloticus× O. aureus*) as influenced by different chromium sources. *The Journal of Nutrition*.

[15] Wang J., Ai Q., Mai K., Xu H., Zuo R. (2014). Dietary chromium polynicotinate enhanced growth performance, feed utilization, and resistance to Cryptocaryon irritans in juvenile large yellow croaker (*Larmichthys crocea*). *Aquaculture*.

[16] Ahmad A. R., Jha A. N., Davies S. J. (2012). The effect of dietary organic chromium on specific growth rate, tissue chromium concentrations, enzyme activities and histology in common carp, *Cyprinus* carpio L. *Biological Trace Element Research*.

[17] Aslam S., Yousafzai A. M. (2017). Chromium toxicity in fish: a review article. *Journal of Entomology and Zoology Studies*.

[18] Cui P., Cheng Z., Sun J. (2022). Effects of different chromium sources on growth performance, serum biochemical, hepatopancreas glycometabolism enzymes activities, IR, GLUT2 and SGLT1 gene expression of common carp (*Cyprinus carpio*). *Aquaculture Research*.

[19] Zakaria M. K., Kari Z. A., Van Doan H. (2022). Fermented soybean meal (FSBM) in African catfish (*Clarias gariepinus*) diets: effects on growth performance, fish gut microbiota analysis, blood haematology, and liver morphology. *Life*.

[20] European Commission (2009). Commission Regulation (EC) No 152/2009 of 27 January 2009 laying down the methods of sampling and analysis for the official control of feed. *Official Journal of the European Union*.

[21] DIN EN 15621 (2017). *Futtermittel - Probenahme- und Untersuchungsverfahren - Bestimmung von Calcium, Natrium, Phosphor, Magnesium, Kalium, Schwefel, Eisen, Zink, Kupfer, Mangan und Cobalt nach Druckaufschluss mittels ICP-AES*.

[22] DIN EN 17053 (2018). *Futtermittel - Probenahme- und Untersuchungsverfahren - Bestimmung von Spurenelementen, Schwermetallen und anderen Elementen in Futtermitteln mittels ICP-MS (Multimethode)*.

[23] Liu T., Wen H., Jiang M. (2010). Effect of dietary chromium picolinate on growth performance and blood parameters in grass carp fingerling, *Ctenopharyngodon idellus*. *Fish Physiology and Biochemistry*.

[24] Ren M., Mokrani A., Liang H. (2018). Dietary chromium picolinate supplementation affects growth, whole-body composition, and gene expression related to glucose metabolism and lipogenesis in juvenile blunt snout bream, *Megalobrama amblycephala*. *Biological Trace Element Research*.

[25] El-Sayed E. H., Hassanein E. I., Soliman M. H., El-Khatib N. R. The effect of dietary chromium picolinate on growth performance, blood parameters and immune status in Nile tilapia, *Oreochromis niloticus*.

[26] Gatta P. P., Piva A., Paolini M. (2001). Effects of dietary organic chromium on gilthead seabream (*Sparus aurata* L.) performances and liver microsomal metabolism. *Aquaculture Research*.

[27] Pan Q., Liu S., Tan Y. G., Bi Y. Z. (2003). The effect of chromium picolinate on growth and carbohydrate utilization in tilapia, *Oreochromis niloticus × Oreochromis aureus*. *Aquaculture*.

[28] Selcuk Z., Tiril S. U., Alagil F. (2010). Effects of dietary L-carnitine and chromium picolinate supplementations on performance and some serum parameters in rainbow trout (*Oncorhynchus mykiss*). *Aquaculture International*.

[29] Shi B., Tao X., Betancor M. B. (2021). Dietary chromium modulates glucose homeostasis and induces oxidative stress in Pacific white shrimp (*Litopenaeus vannamei*). *Aquatic Toxicology*.

[30] Zhang Y., Luo J., Zhu T. (2022). Dietary chromium could improve growth, antioxidant capacity, chromium accumulation in tissues and expression of genes involved into glucose and lipid metabolism in juvenile mud crab *Scylla paramamosain*. *Aquaculture Reports*.

[31] Hardy R. W., Kaushik S. J. (2021). *Fish Nutrition*.

[32] Borel J. S., Majerus T. C., Polansky M. M., Moser P. B., Anderson R. A. (1984). Chromium intake and urinary chromium excretion of trauma patients. *Biological Trace Element Research*.

[33] Chang X., Mowat D. N. (1992). Supplemental chromium for stressed and growing feeder calves. *Journal of Animal Science*.

[34] Moonsie-Shageer S., Mowat D. N. (1993). Effect of level of supplemental chromium on performance, serum constituents, and immune status of stressed feeder calves. *Journal of Animal Science*.

[35] Pechova A., Pavlata L., Illek J. (2002). Metabolic effects of chromium administration to dairy cows in the period of stress. *Czech Journal of Animal Science-UZPI (Czech Republic)*.

[36] Sadoul B., Vijayan M. M. (2016). Stress and growth. *Fish Physiology*.

[37] Jain S. K., Rains J. L., Croad J. L. (2007). Effect of chromium niacinate and chromium picolinate supplementation on lipid peroxidation, TNF-*α*, IL-6, CRP, glycated hemoglobin, triglycerides, and cholesterol levels in blood of streptozotocin-treated diabetic rats. *Free Radical Biology & Medicine*.

[38] Mehrim A. I. (2014). Physiological, biochemical and histometric responses of Nile tilapia (*Oreochromis niloticus* L.) by dietary organic chromium (chromium picolinate) supplementation. *Journal of Advanced Research*.

[39] Tian Y. Y., Zhang L. Y., Dong B., Cao J., Xue J. X., Gong L. M. (2014). Effects of chromium methionine supplementation on growth performance, serum metabolites, endocrine parameters, antioxidant status, and immune traits in growing pigs. *Biological Trace Element Research*.

[40] Lashkari S., Habibian M., Jensen S. K. (2018). A review on the role of chromium supplementation in ruminant nutrition—effects on productive performance, blood metabolites, antioxidant status, and immunocompetence. *Biological Trace Element Research*.

[41] Soengas J. L., Cerdá-Reverter J. M., Delgado M. J. (2018). Central regulation of food intake in fish: an evolutionary perspective. *Journal of Molecular Endocrinology*.

[42] Tran-Duy A., Smit B., van Dam A. A., Schrama J. W. (2008). Effects of dietary starch and energy levels on maximum feed intake, growth and metabolism of Nile tilapia, *Oreochromis niloticus*. *Aquaculture*.

[43] Shiau S. Y., Liang H. S. (1995). Carbohydrate utilization and digestibility by tilapia, *Oreochromis niloticus × O. aureus*, are affected by chromic oxide inclusion in the diet. *The Journal of Nutrition*.

[44] Shiau S. Y., Lin S. F. (1993). Effect of supplemental dietary chromium and vanadium on the utilization of different carbohydrates in tilapia, *Oreochromis niloticus × O. aureus*. *Aquaculture*.

[45] Yuliana Y., Subandiyono S., Hastuti S. (2022). The effect of dietary chromium on growth and survival rate of tilapia (*Oreochromis niloticus*). *Omni-Akuatika*.

